# Supplementary figures and images for: Seasonal patterns of dengue fever in rural Ecuador: 2009-2016
Source: PLoS Negl Trop Dis. 2019 May 6;13(5):e0007360. doi: 10.1371/journal.pntd.0007360 (PMC6522062; doi:10.1371/journal.pntd.0007360)

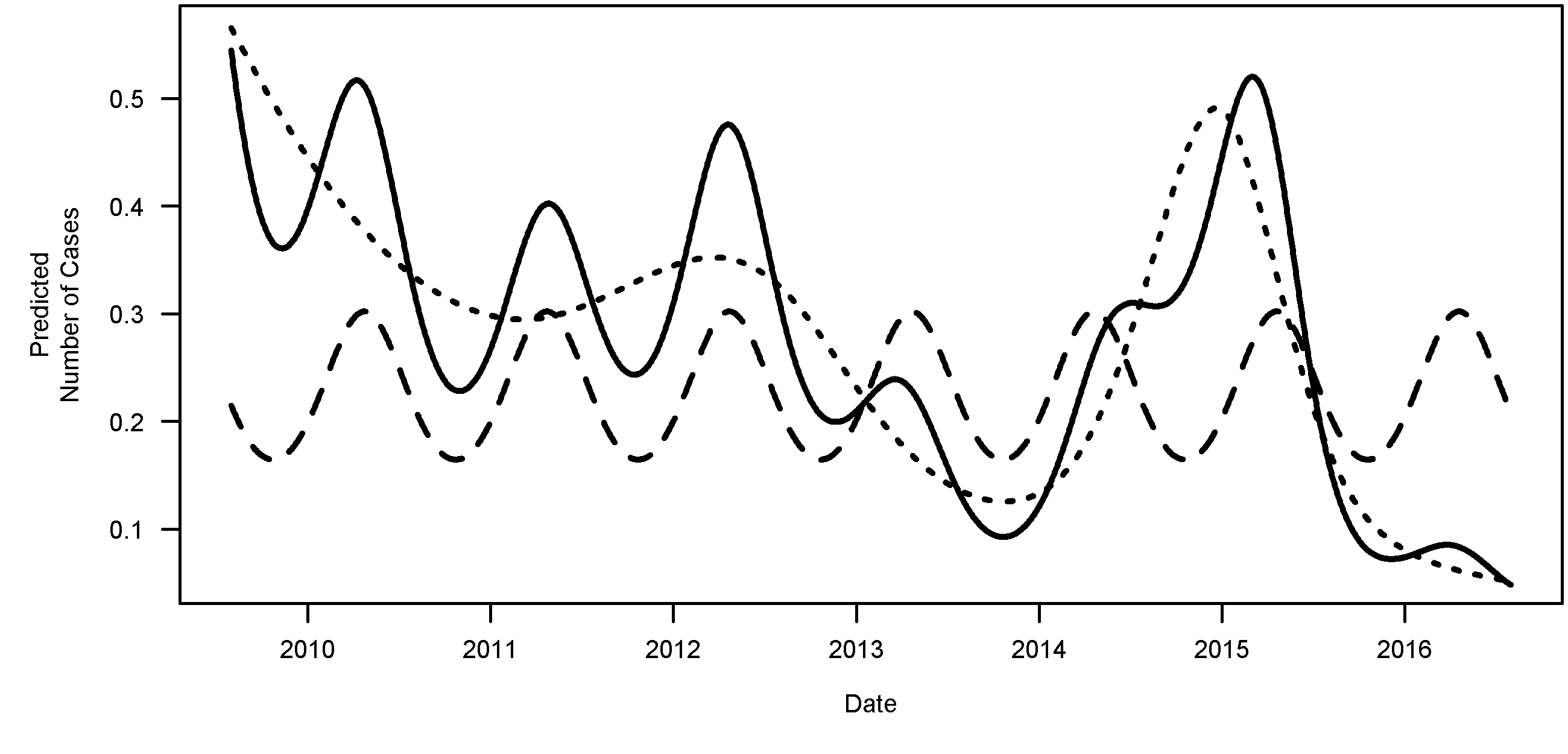

Supplement: S1 Fig — The dashed line is the combined effect of the sine and cosine effects in the model, representing the annual fluctuation of dengue. The dotted line is the effect of the 7-knot spline, representing the long-term or inter-annual fluctuation of dengue. The solid line is the combination of these two effects in Model 1. (TIFF) [file pntd.0007360.s001.tiff]
