# Supplementary material for: Seasonal patterns of dengue fever in rural Ecuador: 2009-2016
Source: PLoS Negl Trop Dis. 2019 May 6;13(5):e0007360. doi: 10.1371/journal.pntd.0007360 (PMC6522062; doi:10.1371/journal.pntd.0007360)
Supplement: S4 Table — Descriptions and metrics for models considered during the analysis of climate seasonality in the dataset. (DOCX) [file pntd.0007360.s005.docx]

| **Model** | **Variables** | **QIC** |
| --- | --- | --- |
| Hospital & temporal | 7-knot spline, sin and cosine (f=365 days), hospital | -1167 |
| Climate variables | 7-knot spline, sin and cosine (f=365 days), hospital, climate variables | -1186 |
| Final model | 7-knot spline, sin and cosine (f=365 days), hospital, climate variables and interactions | -1203 |

QIC=quasi-likelihood under the independence model information criterion
