# Supplementary material for: Seasonal patterns of dengue fever in rural Ecuador: 2009-2016
Source: PLoS Negl Trop Dis. 2019 May 6;13(5):e0007360. doi: 10.1371/journal.pntd.0007360 (PMC6522062; doi:10.1371/journal.pntd.0007360)
Supplement: S5 Table — The variables for Model 1 were applied to a dataset of all infectious disease diagnoses from Hospital Saludesa and Hospital Pedro Vicente Maldonado, resulting in the following effect estimates and 95% confidence intervals. Both effect estimates and 95% confidence intervals have been converted to rate ratios for ease of interpretability. (DOCX) [file pntd.0007360.s006.docx]

| **Parameter** | **Estimate** | **95% Confidence Interval** | | **p-value** |
| --- | --- | --- | --- | --- |
| **Intercept** | 0.58 | 0.47 | 0.71 | <.0001 |
| **sin(2πt)** | 0.92 | 0.88 | 0.97 | 0.0006 |
| **cos(2πt)** | 1.01 | 0.96 | 1.07 | 0.6755 |
| **k^1^** | 1.00 | 1.00 | 1.00 | <.0001 |
| **k1** | 0.92 | 0.89 | 0.94 | <.0001 |
| **k2** | 1.26 | 1.17 | 1.36 | <.0001 |
| **k3** | 0.81 | 0.75 | 0.88 | <.0001 |
| **k4** | 1.08 | 1.04 | 1.12 | 0.0003 |
| **k5** | 0.99 | 0.97 | 1.02 | 0.5928 |
| **Monday^2^** | 1.13 | 1.06 | 1.21 | 0.0002 |
| **Tuesday** | 1.08 | 1.01 | 1.15 | 0.0313 |
| **Wednesday** | 0.99 | 0.93 | 1.07 | 0.8706 |
| **Thursday** | 0.96 | 0.89 | 1.03 | 0.266 |
| **Friday** | 1.01 | 0.94 | 1.08 | 0.8085 |
| **Saturday** | 0.72 | 0.66 | 0.78 | <.0001 |
| **Sunday** | 1.19 | 1.10 | 1.28 | <.0001 |
| **Hospital PVM** | 2.65 | 2.35 | 2.99 | <.0001 |
| **NYE** | 0.70 | 0.51 | 0.96 | 0.026 |
| **An** | 0.90 | 0.58 | 1.39 | 0.6431 |
| **Carnival** | 0.65 | 0.46 | 0.93 | 0.0192 |
| **Easter** | 1.06 | 0.57 | 1.97 | 0.8426 |
| **Labor Day** | 0.46 | 0.27 | 0.77 | 0.0032 |
| **Pichincha** | 1.00 | 0.54 | 1.85 | 0.996 |
| **Independence** | 1.09 | 0.77 | 1.55 | 0.6177 |
| **Guayaquil** | 1.08 | 0.70 | 1.67 | 0.7178 |
| **All Souls** | 0.65 | 0.47 | 0.88 | 0.0063 |
| **Christmas** | 0.57 | 0.28 | 1.20 | 0.1391 |
| **Day after Christmas** | 1.02 | 0.58 | 1.80 | 0.9409 |
